# Effectiveness of drug-coated balloon in acute myocardial infarction: A protocol for systematic review and meta-analysis

**DOI:** 10.1097/MD.0000000000033383

**Published:** 2023-03-31

**Authors:** Aili Yu, Hong Liu, Haiyan Yu, Xue Xiong

**Affiliations:** a Department of Cardiovascular Medicine, Chengdu Eighth People’s Hospital (Geriatric Hospital of Chengdu Medical College), Sichuan, China; b Department of Cardiovascular Medicine, Chengdu Seventh People’s Hospital (Affiliated Cancer Hospital of Chengdu Medical College), Sichuan, China.

**Keywords:** acute myocardial infarction, analysis, coated balloon, drug, drug, eluting stents, meta

## Abstract

The management of acute myocardial infarction (AMI) has improved dramatically over the past 3 decades and is evolving. Percutaneous coronary intervention is an alternative means of achieving coronary revascularization. Previous studies comparing the published literature on drug-coated balloon (DCB) and drug-eluting stents have drawn divergent conclusions. We perform a protocol for systematic review and meta-analysis to compare the efficacy and safety of DCB and drug-eluting stent in the management of AMI.

**Methods:**

This systematic review was registered in the PROSPERO network (registration number: CRD42023397266). We will follow the Preferred Reporting Items for Systematic Reviews and Meta-analysis Protocol to accomplish the systematic review protocol. A systematic search will be conducted in PubMed, Embase, Cochrane Library, Web of Science, Chinese National Knowledge Infrastructure, Wanfang Database, and Weipu Database without any language restrictions from their inception to February 2022. The risk of bias will be assessed independently by 2 authors using parameters defined in the Cochrane Handbook for Systematic Reviews of Interventions criteria. Statistical analysis will be performed using the STATA13.0 software (IBM, USA).

**Results:**

The results of this systematic review and meta-analysis will be publicly available and published in a peer-reviewed journal.

**Conclusion:**

The results of the study will provide the evidence for the application of DCB in the treatment of AMI.

## 1. Introduction

Despite major advancements in primary and secondary prevention strategies, coronary artery disease (CAD) remains a major cause of morbidity and mortality worldwide.^[1–3]^ Percutaneous coronary intervention (PCI), which was introduced as an alternative means of coronary revascularization to coronary artery bypass grafting surgery in 1979,^[4]^ is considered an effective and safe treatment modality for suitable patients with acute or stable CAD. Patients with acute myocardial infarction (AMI) are among the highest-risk patients undergoing PCI. The introduction of stenting decreased the limitations of elastic recoil, restenosis and flow-limiting dissections associated with plain old balloon angioplasty.^[5]^ Due to their improved safety and efficacy compared with first-generation drug-eluting stents and bare-metal stents, new-generation drug-eluting stents are currently recommended for PCI in patients with AMI.^[6,7]^ However, in-stent restenosis, increased risk of bleeding due to prolonged dual antiplatelet therapy, as well as early and late stent thrombosis following implantation may affect the prognosis.^[8,9]^ Furthermore, late stent-related major adverse cardiovascular events occur between 1 to 5 years after PCI,^[10]^ which presents a challenge. For patients with AMI, routine stenting is associated with an increased rate of acute and subacute stent thrombosis compared with stable CAD, and the 1-year incidence of target lesion-related events remains high.^[11]^ In addition, permanent vascular implants impair coronary endothelial and vasomotor functions of the coronary artery.

These limitations resulted in the development of drug-coated balloons. The rationale of drug-coated balloon technology is that a combination of balloons and drugs is used for the treatment of coronary lesions to achieve lower rates of restenosis.^[12]^ Drug-coated balloons (DCB) have emerged as a novel application in PCI, and a DCB strategy has already exhibited successful therapeutic potential for in-stent restenosis and small vessel disease.^[12–14]^ Currently, the use of DCB in AMI remains controversial. Therefore, we perform a protocol for systematic review and meta-analysis to investigate the efficacy and safety of DCB in AMI.

## 2. Methods

This systematic review was registered in the PROSPERO network (registration number: CRD42023397266). We will follow the Preferred Reporting Items for Systematic Reviews and Meta-analysis Protocol to accomplish the systematic review protocol.^[15]^ This study is conducted for the secondary collection and analysis of original data; therefore, ethical approval is not required.

### 2.1. Inclusion criteria

#### 2.1.1. Type of study

All randomized controlled trials on the application of DCBs in the treatment of AMI will be included with no language limitation. However, animal studies, case reports, case series, commentaries, reviews, noncontrolled trials, and other studies that are repeatedly published will be excluded.

#### 2.1.2. Types of participants

Patients who are diagnosed with AMI will be included, without limits on gender, race, nationality, and medical units.

#### 2.1.3. Types of interventions and comparisons

Intervention group receives DCB and control group receives drug-eluting stent (DES).

#### 2.1.4. Types of outcome measures

The primary endpoint is major adverse cardiac events, defined as the composite of cardiac death and target lesion revascularization. The secondary endpoint is late lumen loss, obtained by calculating the difference between the minimum lumen diameter between follow up and post-procedure.

### 2.2. Search methods

A systematic search will be conducted in PubMed, Embase, Cochrane Library, Web of Science, Chinese National Knowledge Infrastructure, Wanfang Database and Weipu Database without any language restrictions from their inception to February 2022. The major search terms are as follows: drug-coated balloon, myocardial infarction, acute coronary syndrome. We also conducted a manual search to confirm the relevant references in the selected articles. The search strategy used for PubMed is presented in Table 1.

**Table 1:** Search strategy for PubMed.

Item	Search terms
#1	Myocardial infarction [Title/Abstract]
#2	Ischemic heart disease [title/abstract]
#3	Acute coronary syndrome [title/abstract]
#4	Coronary artery disease [title/abstract]
#5	Myocardial necrosis [title/abstract]
#6	#1 OR #2 OR #3 OR #4 OR #5
#7	Drug coated balloon [title/abstract]
#8	Drug-eluting balloon [title/abstract]
#9	Paclitaxel-eluting balloons [title/abstract]
#10	Paclitaxel-coated balloons [title/abstract]
#11	DCB [title/abstract]
#12	#7 OR #8 OR #9 OR #10 OR#11
#13	Randomly [title/abstract]
#14	RCT [title/abstract]
#15	Randomized [title/abstract]
#16	#13 OR #14 OR #15
#17	#6 AND #12 AND #16

DCB = drug-coated balloon.

### 2.3. Study selection

**Figure 1.**
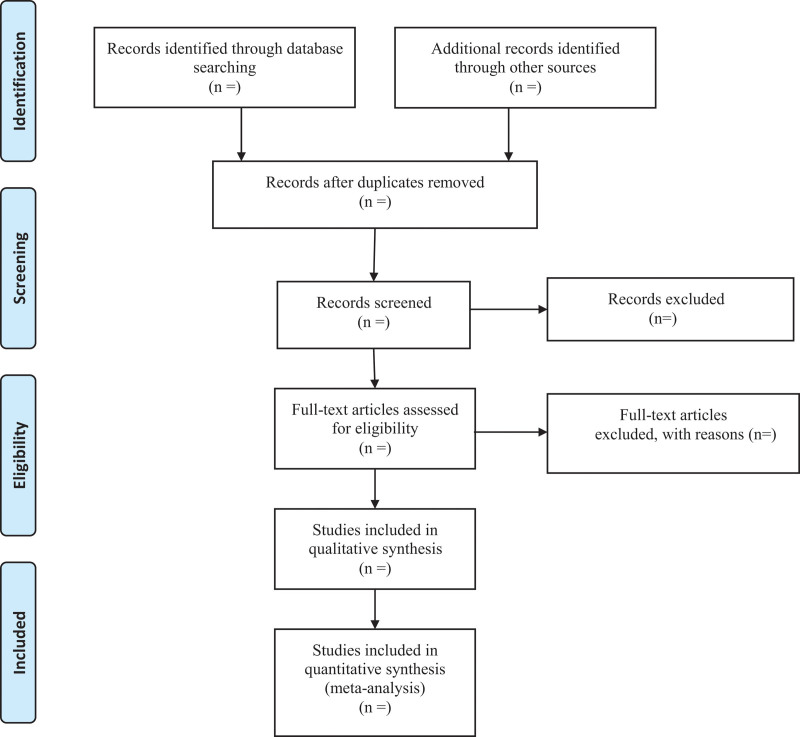
Flow diagram of included randomized controlled trials.

Researchers will discuss and determine the screening criteria within the group before searching the studies. The corresponding research members will import the retrieved studies into the document management system of EndnoteX7 for repetition removal. We will then exclude the apparently unqualified literature by reading the headings and abstracts, and determine the final included literature by reading the full text, discussing within the group and contacting the author to know more about the research details. The final list of included studies will be converted to the format of Microsoft Excel. Both the information retrieval and the literature screening will be independently operated by 2 research members. Finally, another research member will resolve the inconsistency and check the final included studies. Study selection is summarized in a PRISMA flow diagram (Fig. 1).

### 2.4. Data extraction

Data extraction will be conducted by 2 researchers using EpiData 3.1 software (Odense, Denmark) for double entry. Data for collection include age, sample size, disease diagnosis, combined disease, interventions and details about the control group, follow up, outcomes, and adverse event. Any disagreement on data collection will be resolved through discussions or negotiations with the third arbitrator. If the data provided in the study are unclear, missing, or presented in a form that is not extractable or difficult to extract reliably, we will contact the author of the study for clarification.

### 2.5. Risk of bias assessment

The risk of bias will be assessed independently by 2 authors using parameters defined in the Cochrane Handbook for Systematic Reviews of Interventions criteria.^[16]^ The following items will be assessed: random sequence generation (selection bias), allocation concealment (selection bias), blinding (performance bias and detection bias), incomplete outcome data (attrition bias), selective outcome reporting (reporting bias), and other bias. The judgments of evaluated domains will include high, low, and unclear. Disagreements will be resolved by discussion by arbiter.

### 2.6. Statistical analysis

Two researchers respectively enter the data into the STATA13.0 software. Mean differences with a 95% confidence interval are calculated to assess the effect size for continuous outcome data. Risk ratio with a 95% confidence interval are used as effect size for dichotomous data. Inverse variance method and Mantel–Haenszel analysis method are used for continuous variables and dichotomous variables, respectively. The heterogeneity among the trials is assessed for significance with *Q* and quantified with *I*^2^. Statistically significant is set at the *P* value < .10. If the studies are homogeneous or the statistical heterogeneity is low, we use the fixed effect-model. While, random-effects model is applied when the statistical heterogeneity is moderate or high.

### 2.7. Sensitivity analysis

Sensitivity analysis will be used to test the quality of the research contained in the sampled documents. The stability of the conclusions can be tested by reanalyzing the conclusions by inputting missing data and changing the type of research.

### 2.8. Assessment of quality of evidence

The Grading of Recommendations Assessment, Development and Evaluation system will be used to judge the overall quality of evidence supporting outcomes in this work. And the quality of evidence will be defined as high, moderate, low, or very low.

## 3. Discussion

AMI is a common cardiac emergency that can lead to severe morbidity and mortality.^[17]^ The management of AMI has improved dramatically over the past 3 decades and is evolving. Unlike the treatment of older AMI patients, the increased prevalence of AMI in younger people has forced us to pay attention to the long-term risks after stenting, such as lifelong medication, bleeding, etc. DCB can deliver antiproliferative drugs locally without metal support, thereby directly inhibiting the process of endothelial proliferation and negative remodeling.^[18]^ The advantages of treatment with DCB dilation over drug-eluting stents implantation include a lower incidence of restenosis, shorter dual antiplatelet therapy time to reduce the risk of bleeding, and the ability to promote further recovery of endothelial function without leaving any metallic material in the vessels. Previous randomized controlled trials comparing the published literature on DES and DCB have drawn divergent conclusions, as these studies are limited by small sample sizes. To overcome these limitations, we thus conduct a high-quality systematic review and meta-analysis to assess the efficacy and safety of DES versus DCB for patients with AMI.

## Author contributions

**Conceptualization:** Hong Liu.

**Investigation:** Haiyan Yu.

**Methodology:** Haiyan Yu.

**Writing – original draft:** Aili Yu.

**Writing – review & editing:** Xue Xiong.
